# Differential Apoptosis Radiosensitivity of Neural Progenitors in Adult Mouse Hippocampus

**DOI:** 10.3390/ijms17060970

**Published:** 2016-06-20

**Authors:** Yu-Qing Li, Zoey Cheng, Shun Wong

**Affiliations:** 1 Department of Radiation Oncology, Sunnybrook Health Sciences Centre, Toronto, ON M4N 3M5, Canada; yqli@sri.utoronto.ca (Y.-Q.L.); zoey.cheng@mail.utoronto.ca (Z.C.); 2 Institute of Medical Science, University of Toronto, Toronto, ON M5S 1A8, Canada; 3 Departments of Radiation Oncology and Medical Biophysics, University of Toronto, Toronto, ON M4N 3M5, Canada

**Keywords:** neural progenitors, neural stem cells, apoptosis, irradiation, dentate gyrus, p53

## Abstract

Mammalian tissue-specific stem cells and progenitors demonstrate differential DNA damage response. Neural progenitors in dentate gyrus of the hippocampus are known to undergo apoptosis after irradiation. Using a mouse model of hippocampal neuronal development, we characterized the apoptosis sensitivity of the different neural progenitor subpopulations in adult mouse dentate gyrus after irradiation. Two different bromodeoxyuridine incorporation paradigms were used for cell fate mapping. We identified two apoptosis sensitive neural progenitor subpopulations after irradiation. The first represented non-proliferative and non-newborn neuroblasts and immature neurons that expressed doublecortin, calretinin or both. The second consisted of proliferative intermediate neural progenitors. The putative radial glia-like neural stem cells or type-1 cells, regardless of proliferation status, were apoptosis resistant after irradiation. There was no evidence of radiation-induced apoptosis in the absence of the *Trp53* (*p53*) gene but absence of *Cdkn1a* (*p21*) did not alter the apoptotic response. Upregulation of nuclear p53 was observed in neuroblasts after irradiation. We conclude that adult hippocampal neural progenitors may demonstrate differential p53-dependent apoptosis sensitivity after irradiation.

## 1. Introduction

Multipotent neural progenitor cells (NPCs), or stem cells, are present in the adult central nervous system (CNS). They continuously generate new neurons, a process termed neurogenesis [1,2]. An area in adult mammalian brain where neurogenesis has been extensively studied is the dentate gyrus of the hippocampus. Neurogenesis is associated with hippocampal function of learning and memory [3,4,5,6]. Several milestones of neuronal development have been identified. In the prevalent model of adult hippocampal neurogenesis, quiescent radial glial or type-1 cells in the subgranular zone (SGZ) are the putative stem cells. Once activated, they undergo asymmetric divisions to self-renew and generate proliferative type-2 NPCs or intermediate neural progenitors (INPs). INPs in turn give rise to post-mitotic neuroblasts which differentiate into immature, then mature neurons [1,2].

Neurogenesis is a plastic process. Several factors including an enriched environment and exercise are associated with enhanced neurogenesis [7]. Many pathological processes are known to disrupt neuronal development in the adult hippocampus [8]. DNA damage, such as irradiation, is known to inhibit neurogenesis, a process implicated in the neurocognitive decline following cranial radiation treatment [9]. However, it remains unclear how DNA damage response is regulated in NPCs following ionizing radiation, and how this leads to impaired neuronal development in adult CNS [10].

An extensive body of data, primarily *in vitro*, suggests that mammalian stem cells and progenitors in different tissues respond differently to DNA damage [11]. Adult subgranular cells in the dentate gyrus are known to undergo acute apoptosis after irradiation, and this response is mediated by the tumor suppressor gene *p53* [12,13]. However, the relative apoptosis radiosensitivity of the various NPC subpopulations remains unclear. Using the known milestones of neuronal development in adult hippocampus, we show that following irradiation, the apoptosis sensitive subpopulations are the proliferative INPs and non-proliferative neuroblasts and immature neurons whereas the putative radial glia-like neural stem cells regardless of proliferation status are apoptosis resistant. These results are consistent with the notion that during neuronal development in adult hippocampus, p53 mediates distinct DNA damage response in neural stem cells and NPCs to maintain genomic integrity.

## 2. Results

### 2.1. Neuroblasts and Immature Neurons Undergo Radiation-Induced Apoptosis

**Figure 1:**
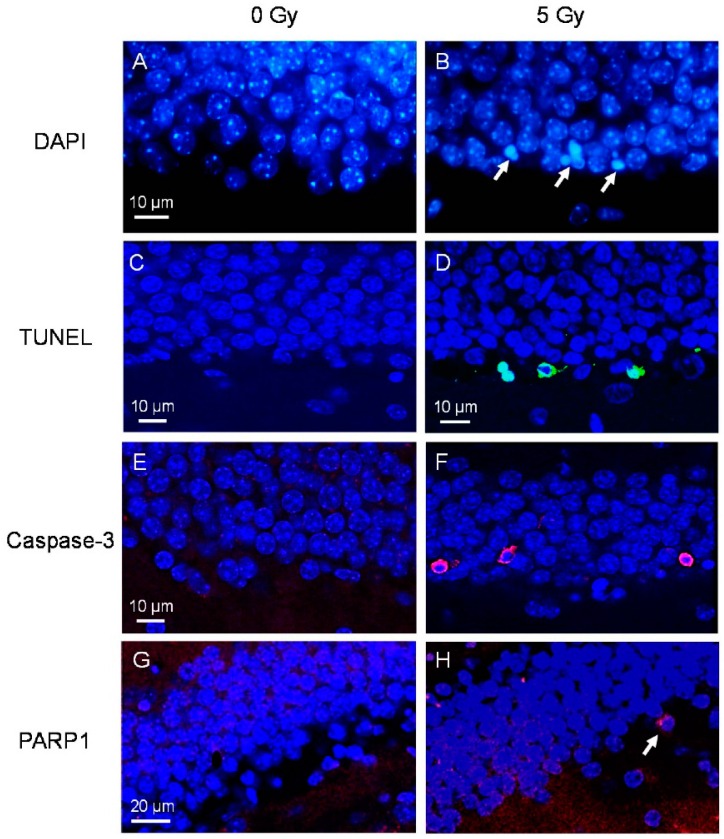
Apoptotic cells in the subgranular zone (SGZ) of dentate gyrus after irradiation. Apoptotic cells are rarely seen in non-irradiated controls (**A**) but are readily seen in SGZ at 8 h after a dose of 5 Gy. They demonstrate characteristic nuclear condensation and fragmentation upon 4′,6-diamidino-2-phenylindole (DAPI) staining ((**B**), arrows). Apoptotic cells are also labeled by terminal deoxynucleotidyl transferase dUTP nick-end labeling (TUNEL) ((**C**,**D**), green), caspase-3 ((**E**,**F**), red) and poly (ADP-ribose) polymerase 1 (PARP1) ((**G**,**H**), red, arrow).

In non-irradiated adult mouse dentate gyrus, apoptotic cells were rarely observed [14]. Within a few hours after irradiation, apoptotic cells could be readily observed in the SGZ. These apoptotic cells demonstrated characteristic condensation and fragmentation of the nucleus following 4′,6-diamidino-2-phenylindole (DAPI) staining (Figure 1A,B). The peak response, 9394.0 ± 1497.2 apoptotic cells in the dentate gyrus, compared to 105.1 ± 23.0 in control (*p* = 0.003, *t*-test), was observed at 8 h after a 5 Gy dose of irradiation. The response returned to non-irradiated control level by 24 h. Of the apoptotic cells identified by characteristic morphology upon DAPI, 83.4% ± 16.1%, 89.1% ± 10.9% and 74.6% ± 3.9% were labeled using terminal deoxynucleotidyl transferase dUTP nick-end labeling (TUNEL), caspase-3 and poly (ADP-ribose) polymerase 1 (PARP1) respectively (Figure 1C–H).

**Figure 2:**
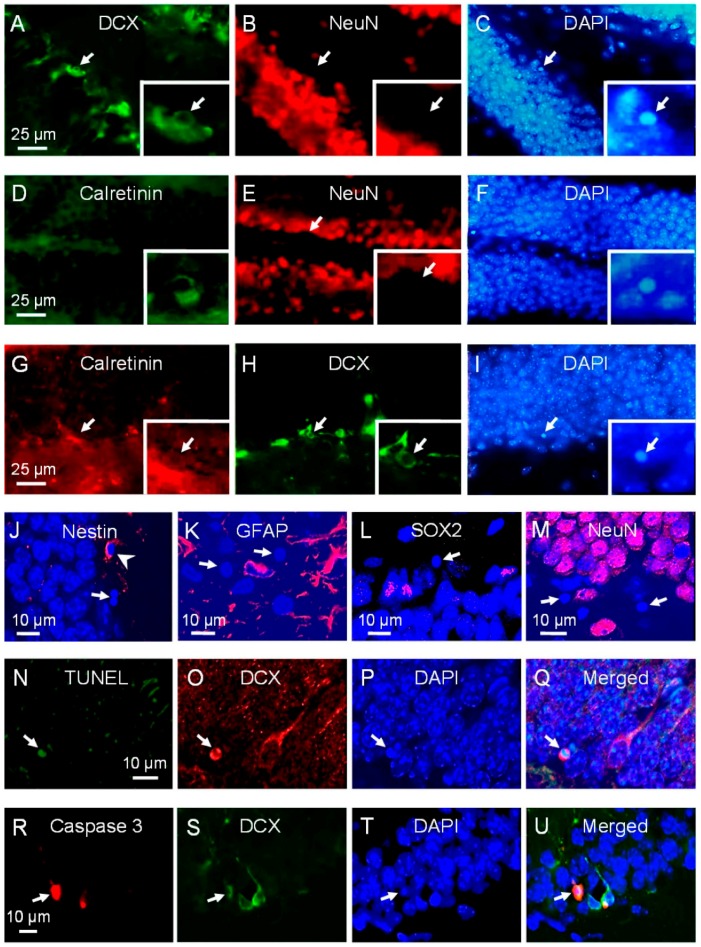
Phenotypic marker expression of apoptotic cells in subgranular zone after irradiation. Apoptotic cells after irradiation demonstrate immunostaining for doublecortin (DCX) and calretinin but not neuronal nuclei (NeuN) ((**A**–**F**), arrows). Some apoptotic cells express both calretinin and DCX ((**G**–**I**), arrows). An occasional apoptotic cell demonstrates immunoreactivity for nestin ((**J**), arrowhead), and apoptotic cells negative for glial fibrillary acidic protein (GFAP) ((**K**), arrows), SOX2 ((**L**), arrow) and NeuN ((**M**), arrows) are observed after irradiation. DCX positive apoptotic cells are identified using TUNEL ((**N**–**Q**), arrow) and caspase-3 immunohistochemistry ((**R**–**U**), arrow).

Apoptotic cells following irradiation have been shown to be NPCs although their exact phenotypes along the milestones of neuronal development remain unclear [10,12,13,15]. Using phenotypic markers singly and in combination for the NPC types described in the SGZ, about a quarter to a third of apoptotic cells after irradiation showed immunoreactivity to doublecortin (DCX), calretinin or both, markers of neuroblasts and immature neurons (Figure 2A–I).

**Figure 3:**
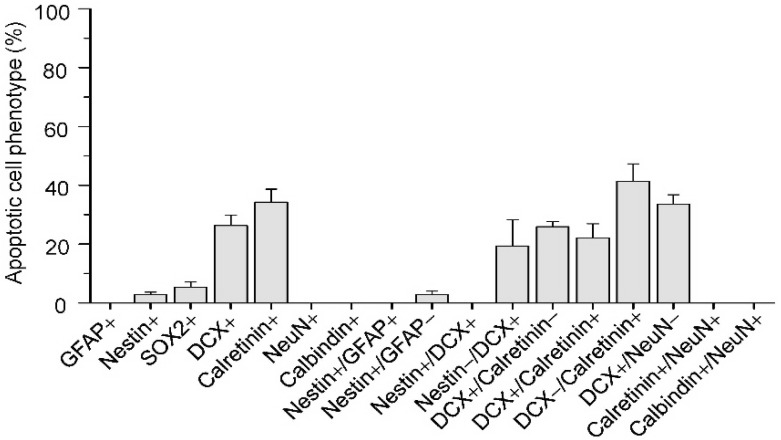
Phenotypes of apoptotic cells in subgranular zone after irradiation. Data represent mean ± standard error, *n* = 3 mice.

We failed to observe any apoptotic cells that were positive for glial fibrillary acidic protein (GFAP), and those that expressed neuronal nuclei (NeuN) or calbindin, markers of late neuronal differentiation and mature neurons. Only occasional apoptotic cells expressed nestin and sex determining region Y-box 2 (SOX2), markers of early NPCs (Figure 2J–M, Figure 3). We did not observe any apoptotic cells positive for DCX or calretinin that expressed nestin or NeuN. Of the TUNEL and caspase-3 positive cells, 39.3% ± 4.3% and 45.1% ± 4.6% respectively expressed DCX (Figure 2N–U). Among the DCX apoptotic cells, 84.4% ± 15.6% were calretinin positive, and of the calretinin positive apoptotic cells, 56.6% ± 17.5% were DCX positive.

**Figure 4:**
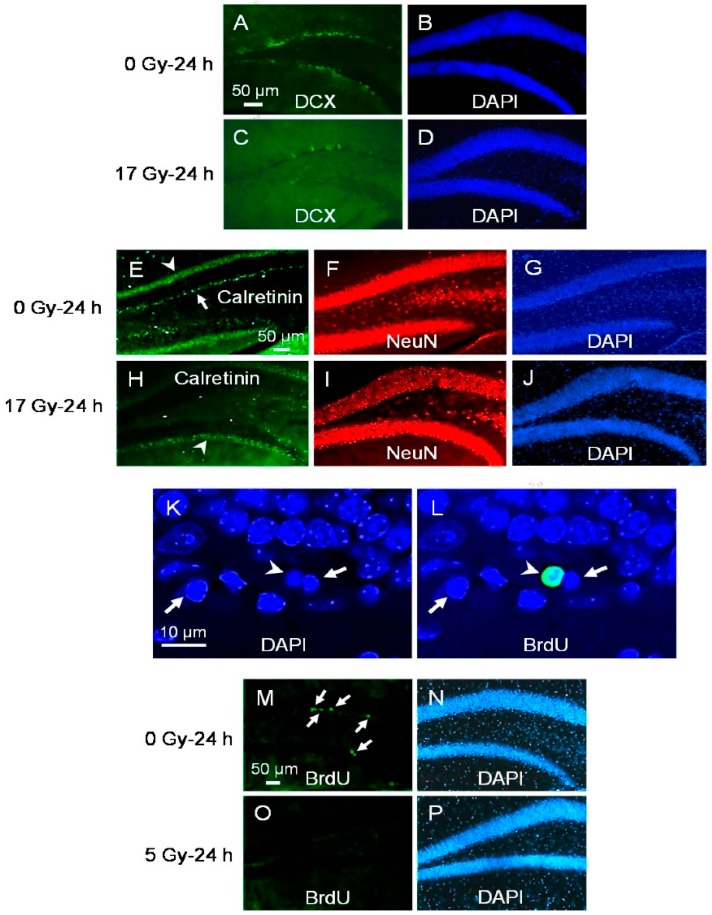
Loss of DCX and calretinin expressing cells, and proliferating cells in subgranular zone (SGZ) at 24 h after irradiation. There is a dramatic loss of DCX positive cells ((**A**–**D**), DCX, green; DAPI, blue) and calretinin positive cells ((**E**–**J**); calretinin cells, arrow, green; NeuN, red; DAPI, blue; arrowhead points to the band of dense calretinin immunoreactive nerve fibers at the inner molecular layer) at 24 h after irradiation. An apoptotic cell in the SGZ demonstrates bromodeoxyuridine (BrdU) incorporation (**K**,**L**), BrdU, green; DAPI, blue; arrowhead) whereas two other apoptotic cells show no BrdU immunoreactivity (arrows); BrdU-retained cells in the SGZ almost disappear completely at 24 h after irradiation ((**M**–**P**), BrdU, arrows, green; DAPI, blue; mice irradiated immediately after BrdU given every 2 h for 4 doses).

The immunogenicity of the phenotypic marker may degrade during the apoptotic process, and the sensitivity of immunohistochemistry may also decrease due to nuclear condensation and fragmentation [13,14]. As a second method to identify the apoptotic radiosensitivity and the early cell fate of the different NPC subpopulations after irradiation, we performed a detailed population analysis of NPCs in the dentate gyrus in controls and at 24 h after 17 Gy. Since the apoptotic response after irradiation is dose-dependent [13], a high dose of 17 Gy was used to induce a large apoptotic response to allow for the detection of changes in cell population. After irradiation, there was a marked reduction in DCX positive (5550.4 ± 2128.0 after 17 Gy compared to 20297.1 ± 2532.6 in control, *p* = 0.01, *t*-test; Figure 4A–D) and calretinin positive cells (309 ± 108, 17 Gy, compared to 2088 ± 1026, control, *p* < 0.05; Figure 4E–J). Cells immunoreactive for both DCX and calretinin almost completely disappeared at 24 h after 17 Gy (Table 1). DCX cells that were either nestin positive or nestin negative were both significantly reduced at 24 h after irradiation (Table 1).

**Figure 5:**
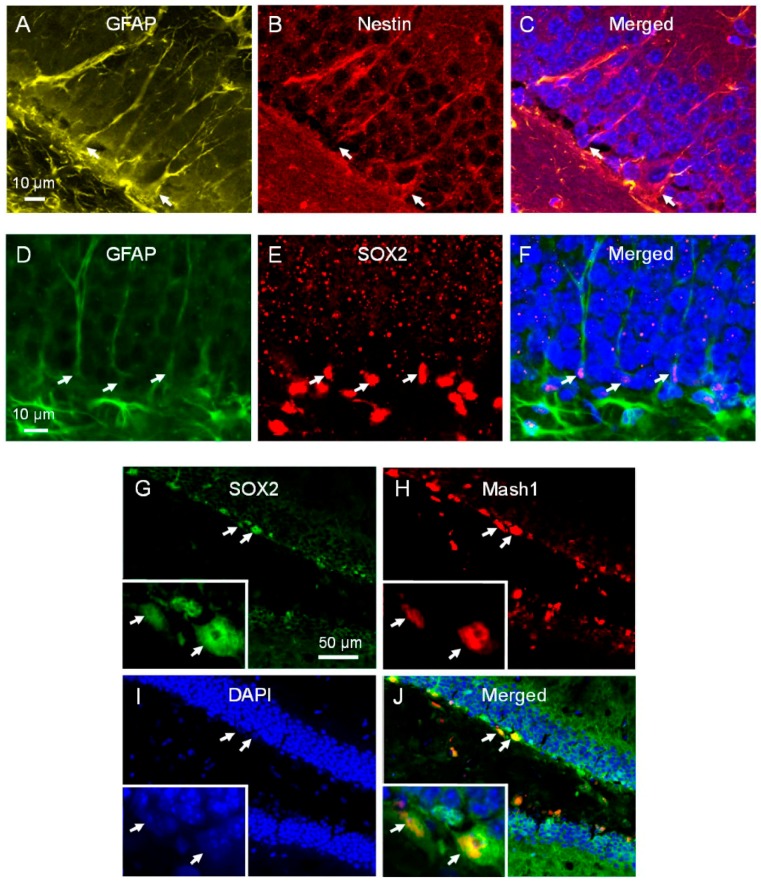
Type-1 and type-2 cells in subgranular zone of dentate gyrus. Radial glial or type-1 cells (**A**–**F**, arrows) in dentate gyrus express GFAP ((**A**), arrows, yellow) and nestin ((**B**), red; (**C**), merged), or GFAP ((**D**), arrows, green) and SOX2 ((**E**), red; (**F**), merged); type-2 cells express SOX2 ((**G**), arrows, green) and Mash1 ((**H**), arrows, red; (**I**), DAPI, arrows, blue; (**J**), arrows, merged) and have short processes.

The putative neural stem cells in the dentate gyrus are the radial glial cells, also known as type-1 cells. They have a triangular cell body and a long radial process that spans the entire granule cell layer and ramifies in the molecular layer. Type-1 cells express GFAP, nestin and SOX2 [16]. After irradiation, we observed no change in the number dual GFAP/nestin positive cells and GFAP/SOX2 positive cells that demonstrated the characteristic morphology of type-1 cells at 24 h (Figure 5A–F, Table 1). There was also no significant reduction in the number of nestin-positive/DCX-negative cells and dual SOX2/Mash1 positive cells (Figure 5G–J), phenotypes of early INPs or type-2a cells at 24 h after irradiation (Table 1).

These results of the immunohistochemistry and cell population analysis suggest that the majority of the apoptosis radiosensitive NPCs are type-2b (late INPs), type-3 (neuroblasts) and calretinin-positive immature neurons. Neural stem cells or type-1 cells appear to be resistant to radiation-induced apoptosis.

### 2.2. Proliferating Early NPCs but Not Newborn NPCs Undergo Radiation-Induced Apoptosis

During neurogenesis in adult dentate gyrus, only a few newborn cells become mature neurons and integrated into the hippocampal circuitry. The majority of newborn cells are thought to undergo apoptosis [14]. We therefore asked whether newborn cells might be particularly susceptible to apoptosis after irradiation. For this study, animals were given an intraperitoneal (ip) injection of bromodeoxyuridine (BrdU), 50 mg/kg/day daily for 7 days. They then received a single 5 Gy one day after the last BrdU injection. Using these 7 days of BrdU administration paradigm, the majority of BrdU labeled cells were expected to be newly divided cells. At 8 h after irradiation, only 5.3% ± 2.7% of the subgranular apoptotic cells demonstrated BrdU incorporation (Figure 4K,L). Further, the number of BrdU retained cells in dentate gyrus at 24 h after irradiation remained unchanged compared to non-irradiated controls (729.2 ± 35.8, 5 Gy *vs.* 718.5 ± 17.9, 0 Gy, *p* not significant, *t*-test). Hence, the majority of apoptotic radiosensitive neuroblasts or immature neurons were unlikely to be newly born during the week prior to irradiation.

To determine whether proliferating status of the NPCs influence their apoptosis radiosensitivity, animals were given BrdU (50 mg/kg) every 2 h for 4 doses, and were given a single 5 Gy dose immediately after the last BrdU injection. Mice were sacrificed at 24 h after irradiation. Using this BrdU administration paradigm, the majority of BrdU labeled cells represented proliferating cells. A dramatic clearance of BrdU labeled cells was apparent after 5 Gy (Figure 4M–P). The total number of BrdU labeled cells at 24 h decreased dramatically after irradiation (332.5 ± 18.2 compared to control, 3205.4 ± 323.4, *t*-test *p* < 0.001). The total number nestin/BrdU positive cells at 24 h also decreased after irradiation (280.0 ± 20.2 compared to control, 3138.3 ± 341.9, *p* < 0.001). There was no change in the number of type-1 (GFAP/nestin positive) cells that demonstrated BrdU incorporation (52.5 ± 15.1 compared to control, 67.1 ± 19.1, *p* not significant, *t*-test). These results identified proliferating INPs as a second population of cells that were ablated within 24 h after irradiation. Neural stem cells regardless of their proliferating status were resistant to radiation-induced apoptosis.

### 2.3. There Is Differential Activation of p53 in NPCs after Irradiation

**Figure 6:**
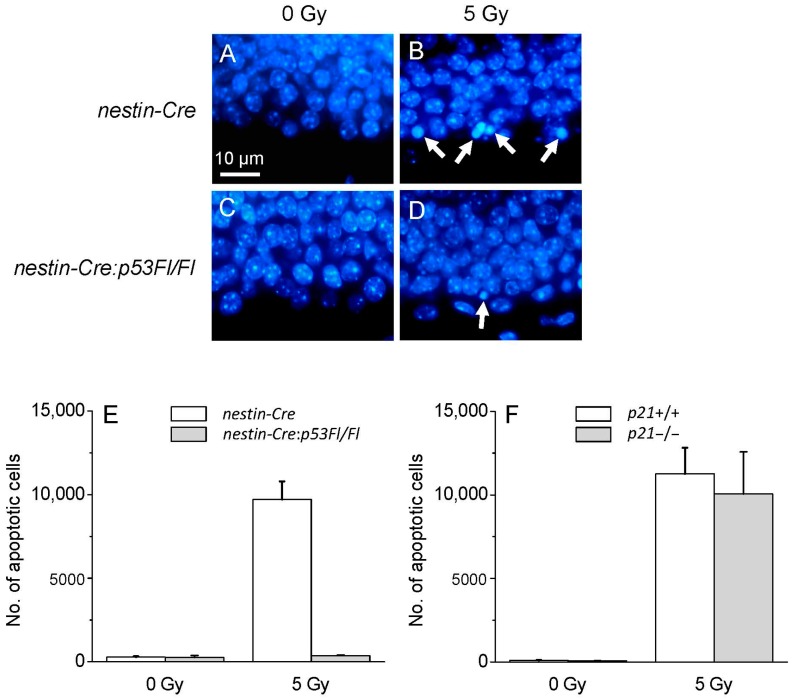
Radiation-induced apoptosis is abrogated in p53 deficient mice but not altered in p21 deficient mice. A robust apoptotic response is observed in subgranular zone 8 h after 5 Gy in *nestin-Cre* mice but no significant response is observed in *nestin-Cre:p53Fl*/*Fl* mice (**A**–**E**); *p21* genotype status does not alter the apoptotic response (**F**). *n* = 3 mice per dose per genotype. Apoptotic cells, arrows.

Radiation-induced apoptosis of subgranular cells is known to be p53 dependent [12,13,17]. It was extremely difficult to observe apoptotic cells in SGZ of *p53*−/− mice after irradiation (data not shown). A disrupted microenvironment has been shown to modify the apoptotic response of NPCs after irradiation [13]. To further determine the role of p53 in radiation-induced NPCs in the dentate gyrus, *nestin-Cre:p53Fl*/*Fl* mice with targeted *p53* disruption in nestin expressing cells only were given either 0 or 5 Gy, and the apoptotic response was assessed 8 h after irradiation. There was no evidence of an apoptotic response in the SGZ of *nestin-Cre:p53Fl*/*Fl* mice whereas a robust response was observed in *nestin-Cre* mice which served as controls (irradiation, *p* < 0.001; *p53Fl*/*Fl* genotype, *p* < 0.001; interaction, *p* < 0.001; 2-way ANOVA; Figure 6A–E).

**Figure 7:**
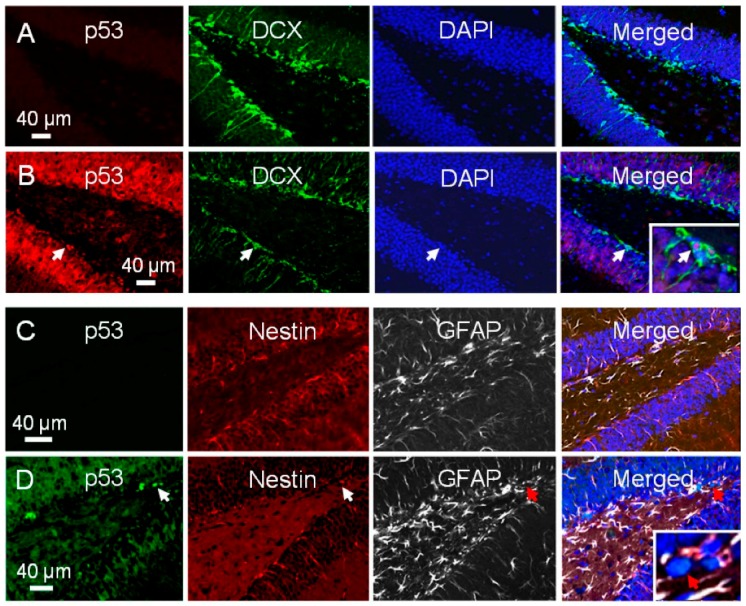

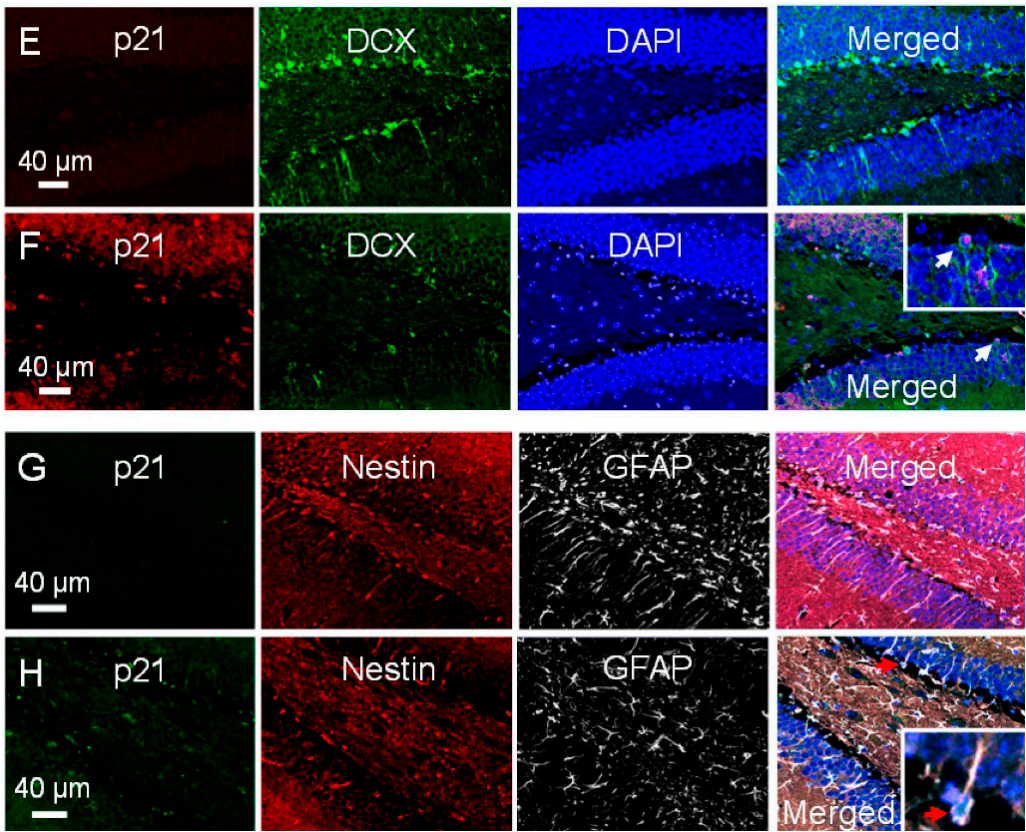
There is upregulation of p53 and p21 in dentate gyrus after irradiation. There is no evidence for p53 immunoreactivity in dentate gyrus of non-irradiated mice (**A**); after irradiation, p53 nuclear immunoreactivity is seen in dentate gyrus at 8 h ((**B**), p53, red; DCX, green; DAPI, blue), and some p53 immunoreactive cells are DCX positive ((**B**), merged, arrow, inset); cells immunoreactive for p53 do not express nestin or GFAP ((**C**), control; (**D**), 5 Gy; p53, green; nestin, red; GFAP, white; DAPI, blue; arrows); there is no p21 immunoreactivity observed in non-irradiated dentate gyrus; increase in p21 nuclear immunoreactivity is seen at 8 h after irradiation ((**E**), 0 Gy; (**F**), 5 Gy; p21, red; DCX, green; DAPI, blue; (**G**); 0 Gy; (**H**), 5 Gy, p21, green; nestin, red; GFAP, white; DAPI, blue). Some cells immunoreactive for p21 express DCX ((**F**), merged, arrow, inset), and some express both nestin and GFAP ((**H**), merged, arrow, inset).

We asked whether there was differential upregulation of p53 associated with the differential apoptotic response among the NPCs after irradiation. In non-irradiated dentate gyrus, there was no evidence of p53 immunoreactivity (Figure 7A). At 8 h after 5 Gy, there was a generalized increase in p53 nuclear immunoreactivity in dentate gyrus, but some subgranular cells that expressed DCX demonstrated intense p53 nuclear staining (Figure 7B). Among the p53 positive subgranular cells, 79.0% ± 2.3% showed immunoreactivity for DCX whereas only 4.3% ± 0.4% showed dual nestin/GFAP immunoreactivity (Figure 7B–D). Of the DCX positive cells, 67.2% ± 2.3% demonstrated p53 nuclear immunoreactivity after irradiation, whereas only 7.9% ± 0.9% of GFAP/nestin positive cells with type-1 cell morphology demonstrated p53 nuclear immunoreactivity after irradiation. Hence, most cells that demonstrated p53 upregulation were DCX expressing neuroblasts and not the putative neural stem cells. There was no evidence of p53 immunoreactivity seen in control or irradiated *p53*−/− mouse hippocampus which served as negative control.

### 2.4. Radiation-Induced Apoptosis Is Not Influenced by p21 Status

Irradiation is known to activate p53 and upregulate the cell cycle inhibitor p21, a known downstream effector of p53. We asked whether the apoptotic response in dentate gyrus is altered in the absence of p21. Mice wild type or knockout for the *Cdkn1a* (*p21*) gene were given a dose of 0 or 5 Gy. At 8 h after irradiation, the apoptotic response after irradiation was similar in *p21*−/− mice compared to *p21*+/+ animals (irradiation, *p* < 0.001; *p21* genotype, not significant, 2-way ANOVA; Figure 6F).

There was no evidence for p21 immunoreactivity in non-irradiated mouse hippocampus. At 8 h after 17 Gy, increase in p21 nuclear immunoreactivity was observed in dentate gyrus including cells in the SGZ. Among the DCX positive cells, 29.9% ± 3.7% demonstrated p21 nuclear immunoreactivity after irradiation (Figure 7E,F), and 19.4% ± 2.7% of type-1 cells were p21 positive (Figure 7G,H). Among the p21 positive subgranular cells, 29.0% ± 3.7% showed immunoreactivity for DCX and 30.3% ± 5.4% showed dual nestin/GFAP immunoreactivity and morphology for type-1 cells. Hence, there was no evidence for differential upregulation of p21 in irradiated neuroblasts *versus* type-1 cells in mouse hippocampus. Immunoreactivity for 21 was not observed in *p21*−/− mice, which served as negative control.

## 3. Discussion

During neurogenesis in adult dentate gyrus, only a few newborn cells become mature neurons and integrate into the hippocampal circuitry. The majority of newborn cells are thought to undergo apoptosis. The apoptotic cells were shown to be late INPs before they transition into neuroblasts [14]. Here, we showed that the majority of apoptotic cells after irradiation were neuroblasts and immature neurons that expressed DCX, calretinin or both. Results of the population analysis showed a marked depletion of cells immunoreactive for DCX, calretinin, or dual DCX/calretinin at 24 h after irradiation, and were consistent with results based on phenotypic marker expression in apoptotic cells. Using a BrdU administration paradigm to label newborn cells, we further revealed that the apoptosis radiosensitive cells were not neuroblasts or immature neurons born within the week prior to irradiation. These results were in contrast to normal neurogenesis during which most of the apoptotic cells are INPs born within the first few days of life [14].

The marked clearance of BrdU/nestin labeled cells at 24 h after multiple BrdU injections before irradiation identified proliferating INPs as a second subpopulation of NPCs that undergo apoptosis after DNA damage. The early loss of proliferating INPs after irradiation due to apoptosis is supported by the observation that about 5% of the apoptotic cells also expressed nestin or SOX2. Although BrdU is known to be a radiosensitizer, it is highly unlikely that INPs only become apoptosis radiosensitive following BrdU incorporation. Radiosensitization also requires prolonged drug administration to allow high percentage of replacement of thymidine bases with BrdU [18]. A previous study [15] also showed that irradiation resulted in a marked decrease in Ki67 labeled cells at 48 h, and concluded that the proliferative pool of NPCs represented the apoptosis sensitive subpopulation of cells. However this could have been related to cell cycle effects after irradiation. It is also unlikely though possible that the early loss of proliferating IPCs after irradiation could be due to other modes of cell death, such as division coupled non-apoptotic death.

There is compelling evidence from genetic ablation and transgenic fate mapping studies that GFAP expressing radial glia-like cells or type-1 cells are the neural stem cells required for constitutive adult neurogenesis [16,19,20]. Fate mapping studies revealed that a single radial glia-like cell upon exiting their quiescent state undergoes only two to three rounds of asymmetric divisions to produce mature neurons and self-renew [16]. During normal adult neuronal development in the hippocampus, division coupled production of new neurons is believed to result in depletion of the adult neural stem cell pool [21]. The present results provide no evidence that type-1 cells or neural stem cells, regardless of whether they are quiescent or proliferating, are susceptible to early apoptotic death or other modes of cell death within 24 h after irradiation. We also showed that immature neurons by the time they expressed NeuN became resistant to apoptosis after irradiation.

Most data on DNA damage response of tissue-specific stem cells are based on *in vitro* observations and considerable heterogeneity in response has been observed [10]. Irradiation is known to activate p53 and upregulate the cell cycle inhibitor p21, a known downstream effector of p53. The DNA damage response of hematopoietic and mammary stem cells *in vitro* was found to result in p21 upregulation and inhibition of p53 basal activity. It has been postulated that this resulted in inhibition of apoptosis and acquisition of symmetric self-renewal divisions after irradiation [22]. We showed that the majority of type-1 cells did not demonstrate p53 upregulation after irradiation, whereas increased p53 nuclear immunoreactivity was observed in neuroblasts after irradiation.

Both p53 and p21 are known to contribute to the relative quiescence of neural stem cells and regulate adult neurogenesis [23,24,25,26,27]. In the developing mouse cortex, post-mitotic migrating cortical cells demonstrated a p21 dependent apoptotic response after in-utero exposure to ionizing radiation. Lack of activation of a cell cycle checkpoint and apoptosis of post-mitotic migrating cells after DNA damage appeared to depend on the expression of p21 [28]. Here, the absence of *p21* gene did not result in any difference in the apoptotic response of NPCs in the adult SGZ. This observation is unlikely to be confounded by a difference in the proliferating INP population in *p21*−/− mice since p21 loss was not shown to alter neural progenitor cell populations in young adult mice based on Ki67- and BrdU-labeling [23]. A small number of type-1 cells demonstrated p21 upregulation after irradiation. Given that type-1 cells are apoptosis resistant after irradiation, p21 upregulation is unlikely to be associated with the apoptotic process. Our results are rather consistent with the lack of a significant role of p21 in regulating the apoptotic response in adult dentate gyrus in response to DNA damage after irradiation.

In summary, there are two apoptosis sensitive NPC subpopulations in adult dentate gyrus after irradiation. The first represents post-mitotic, lineage determined, non-newborn neuroblasts and early immature neurons. There is a second apoptosis radiosensitive subpopulation of NPCs, the proliferating nestin expressing INPs. Neural stem cells or type-1 cells, regardless of their proliferating status, do not undergo apoptosis in response to DNA damage after irradiation. These findings suggest that NPCs in adult dentate gyrus have differential p53 dependent apoptotic response after DNA damage.

## 4. Materials and Methods

### 4.1. Animals

C57 mice, wild type (+/+) or knockout (−/−) for the *Trp53* (*p53*) gene, and C57 mice +/+ or −/− for the *Cdkn1a* (*p21*) gene were originally obtained from the Jackson Laboratory (Bar Harbor, ME, USA). Ten-week-old male *p53*+/+, *p53*−/−, *p21*+/+ and *p21*−/− were used in this study. Genotyping was performed by PCR.

Mice with targeted *p53* disruption in nestin expressing cells only were used to determine the role of p53 in radiation-induced apoptosis in NPCs. *Nestin-Cre* mice were kind gifts from Dr. A. Nagy. These mice have CD1 background and possess the *Tg(Nes-cre)1Kln* transgene construct [29]. They were bred with *Trp53^tm1Brn^* (*p53Fl*) mice (The Jackson Laboratory, stock No. 008462) [30] of C57 background, to obtain mice with both *nestin-Cre* and *p53Fl* transgenes. *Nestin-Cre*:*p53Fl/*. mice were bred with *p53Fl*/. mice or *p53Fl*/*Fl* mice to obtain *nestin-Cre:p53Fl*/*Fl* mice, which were used in the experiments. Hemizygous *nestin-Cre* mice served as controls. All *nestin-Cre:p53Fl*/*Fl* mice and *nestin-Cre* mice had mixed C57 and CD1 background. The presence of the transgenes was confirmed with PCR. The primer sequences to detect *Cre* transgene were: forward, 5′-GCGGTCTGGCAGTAA AAACTATC-3′; reverse, 5′-GTGAAACAGCATTGCTGTCACTT-3′; internal positive control forward, 5′-CTAGGCCACAGAATTGAAAGATCT-3′; and internal positive control reverse, 5′-GTAGGTGGAAATTCTAGCATCATCC-3′. The primer sequences to detect *p53Fl* were: forward, 5′-GGTTAAACCCAGCTTGACCA-3′; reverse, 5′-GGAGGCAGAGACAGTTGGAG-3′.

Mice were housed under a 12-h light/12-h dark cycle at 21 °C, with food and water *ad libitum*. Only male mice were used in all the experiments to avoid the potential confounding influence of gender differences on radiation responses [31]. There was a minimum of 3 mice per genotype per dose per time point. All animal protocols were evaluated and approved by the Animal Care Committee, Sunnybrook Research Institute (Animal Use Protocol No. 156), and experiments were performed according to the guidelines set by the Canadian Council on Animal Care.

### 4.2. Irradiation

Mice were anesthetized with an intraperitoneal injection of a cocktail of ketamine (75 mg/kg) and xylazine (6 mg/kg), and immobilized in a customized jig during whole brain irradiation using 160 kV X-rays, as previously described [13].

### 4.3. Bromodeoxyuridine (BrdU) Incorporation

Two different BrdU administration paradigms were used for cell fate mapping, as described in Results.

### 4.4. Histopathology and Immunohistochemistry

Mice were perfused with 0.9% saline, followed by 4% paraformaldehyde in PBS under anesthesia using ketamine and xylazine as described [13]. Mouse brains were retrieved, post-fixed for 2 days, and cryoprotected in a 30% sucrose solution. Coronal sections of the hippocampus between −1.34 mm and −3.08 mm, relative to the bregma [32] were cut at 40-μm thickness. Sections were stored at −20 °C in tissue cryoprotectant solution in 96-well plates as previously described [13].

Cells that showed chararcteristic nuclear condensation and fragmentation upon DAPI staining were considered apoptotic cells as we previously described [13,14]. Selected results based on DAPI were confirmed using TUNEL, caspase-3, and PARP1 staining. Methods for TUNEL staining were as we previously described [13]. Rabbit anti-caspase-3 (1:1000; Cell Signaling Technology, Danvers, MA, USA), rabbit anti-PARP1 (1:200; Cell Signaling Technology) and donkey anti-rabbit Cy3 secondary antibody (1:200; Jackson ImmunoResearch, West Grove, PA, USA) were used for caspase-3 and PARP1 immunohistochemistry. DAPI counterstaining was used to identify nuclear morphology of apoptosis.

Free floating sections were washed in PBS, incubated with the different phenotypic markers for NPCs, immature and mature neurons. The primary antibodies used were listed in the Supporting Information Table. Secondary antibodies conjugated to Cy2 (1:200; Jackson ImmunoResearch), Cy3 (1:200; Jackson ImmunoResearch), or Alexa Fluor 647 (1:200; Invitrogen, Burlington, Ontario, Canada) were used to reveal the immunoreactivity. Sections were counterstained with DAPI. Morphology was also used to identify type-1 and type-2 cells. Cells that were dual-stained for GFAP and nestin, and have a radial process spanning the granule cell layer which ramifies in the inner molecular layer, were considered type-1 cells. Cells in the SGZ that were nestin positive but GFAP negative, and had short processes only, were considered type-2 cells. A confocal laser scanning microscope (Zeiss LSM700; Oberkochen, Germany) was used to evaluate co-localization of BrdU and the various phenotypic markers in selected sections.

### 4.5. Stereological Analysis

Cell counting was performed within the dentate gyrus including a 50-µm hilar margin of the SGZ, as previously described [13], using a Zeiss Imager M1 microscope with the Stereo Investigator software (MicroBrightField, Williston, VT, USA). The observer was blinded to the treatment. Cells were counted using a counting frame and a sampling grid of 75 µm × 75 µm at a magnification of 63×. NPCs were counted using a counting frame of 20 µm × 20 µm and a sampling grid of 180 µm × 180 µm. All other stereologic parameters were otherwise the same. Every seventh section was used as the periodicity of sections sampled.

The estimated cell number represented the mean of the number of the target cells in the right and left dentate gyrus. Completeness of immunostaining throughout the entire thickness of the section was reviewed prior to cell counting. For all stereological counting, the coefficient of error was between 0.03 and 0.06 in.

### 4.6. Statistical Analysis

All data represented the mean ± standard error. Differences between control and irradiated mice were evaluated using *t*-test. The influence of irradiation and genotype on the apoptotic response was determined using two-way ANOVA. A *p* value < 0.05 was considered statistically significant. Statistical analyses were performed with the GraphPad Prism 4 (GraphPad Software, San Diego, CA, USA).

## Figures and Tables

**Table 1:** Changes in neural progenitor and neuronal populations in mouse dentate gyrus at 24 h after irradiation.

Cell Type (Phenotypic Markers)	Number of Cells	
	0 Gy	17 Gy
Type 1, neural stem cells (nestin+/GFAP+)	1826 ± 178	1887 ± 248
Type 1, neural stem cells (SOX2+/GFAP+)	980 ± 84	831 ± 252
Type 2a, early INPs (nestin+/DCX−)	1782 ± 392	1222 ± 340
Type 2a, early INPs (SOX2+/Mash1+)	2228 ± 354	1753 ± 564
Type 2b, late INPs (nestin+/DCX+)	650 ± 121	265 ± 53 *
Type 3, neuroblasts (nestin+/DCX+)	15,517 ± 240	3646 ± 56 **
Immature neurons (DCX+/calretinin+)	1476 ± 1075	3 ± 3 **
Mature neurons (NeuN+/calbindin+)	37,112 ± 2980	48,347 ± 11,897
Mature neurons (NeuN+)	428,274 ± 38,591	393,498 ± 25,634

* *p* < 0.05; ** *p* < 0.01 (*t*-test, compared to 0 Gy); *n* = minimum of 3 mice; INPs = intermediate neural progenitors.

## Supplementary Materials

Supplementary materials can be found at http://www.mdpi.com/1422-0067/17/6/970/s1.
