# Supplementary material for: Differential Apoptosis Radiosensitivity of Neural Progenitors in Adult Mouse Hippocampus
Source: Int J Mol Sci. 2016 Jun 20;17(6):970. doi: 10.3390/ijms17060970 (PMC4926502; doi:10.3390/ijms17060970)
Supplement: Supplementary file 1 [file ijms-17-00970-s001.pdf]

Supplementary Materials: Differential Apoptosis Radiosensitivity of Neural Progenitors in Adult Mouse Hippocampus

Yu-Qing Li, Zoey Cheng and Shun Wong

Table S1. List of primary antibodies used.

Antibodies	Dilution	Company
BrdU	1:200	Abcam
Caspase-3	1:1000	Cell Signaling Technology
Calbindin	1:1000	Millipore
Calretinin	1:200	Abcam
DCX	1:2000	Abcam
GFAP	1:200	DakoCytomation
Mash1	1:50	Abcam
Nestin	1:200	Millipore
NeuN	1:500	Millipore
p21	1:50	Abcam
p53	1:500	Novocastra
PARP1	1:200	Jackson ImmunoResearch
SOX2	1:150	Abcam

Abbreviations: BrdU, bromodeoxyuridine; DCX, doublecortin; GFAP, glial fibrillary acidic protein; NeuN, neuronal nuclei; PARP1, poly (ADP-ribose) polymerase 1; SOX2, sex determining region Y-box 2.
